# A bridge between worlds: Embedding research in telepractice co‐design with disability community

**DOI:** 10.1002/lrh2.10428

**Published:** 2024-05-10

**Authors:** Cloe Benz

**Affiliations:** ^1^ School of Population Health, Faculty of Health Sciences Curtin University Bentley Western Australia Australia

**Keywords:** co‐design, disability, embedded research

## Abstract

**Introduction:**

Co‐production approaches are increasingly being advocated for as a way of addressing the research translatory gap while including patient and public involvement in development of services they access, and particularly in disability service provision. Embedded research (ER) is a method which integrates the researcher within the target organization to better facilitate both co‐production of research outputs and the reduction of the research translation gap. The aim of this reflection is to better understand the commonalities and differences between ER in a disability context to accounts published in academic literature.

**Method:**

A review of embedded researcher literature was completed in combination with a personal reflection of lived experience as an embedded researcher within a disability support service organization. The reflective process included review of research journal entries and other records of lived experience (photographs, audio recordings, drawings) maintained throughout the period in the role of embedded researcher. A reflexive thematic analysis process was used.

**Results:**

I reflect throughout the article upon five themes which highlight both the commonalities between my experiences and those of other embedded researchers as well as instances where they differed. The five themes include (1) A knowledge bridge, (2) Considerations of positionality, (3) Ethical complexity, (4) Anticipating change, and (5) Existing in the in‐between together.

**Conclusion:**

Experiences of ER appear to transcend the discipline in which the research is being embedded, and while the lived experience in a disability host organization was invaluable in facilitating a successful co‐produced research project, significant avenues for improvement exist in terms of ethical frameworks, methodological guidance, and communities of support.

## 1 INTRODUCTION

Embedded research (ER) literature frequently draws methodological justification through limiting the research‐to‐practice translation gap,^1^ by integrating researchers and co‐producing research within organizations. Value in reducing the translation gap has been demonstrated in a variety of contexts including climate science,^2^ public health,^1^ child protection,^3^ education,^4^ medicine,^5^ and immunization.^6^, ^7^ However, this is the first paper to my knowledge which focuses on the experience of ER in disability and social care settings.

Repeated calls exist both in academic literature^8^ and by prominent disability advocates^9^ to elevate the involvement of people with disability and their families in decision‐making for areas impacting them. In order to facilitate inclusive practices such as community‐based participatory research,^10^ there is a need for researchers to assimilate into the disability community to understand their needs and tailor research enabling them to participate in co‐production.^11^ Roles that bridge the gap between community, providers, and research have the potential to support the growth of partnerships and collaboration. The ER role has been described as occupying the space in‐between research and practice.^12^ An alternate bridging role is a peer researcher, who occupies the space in‐between community and research, in this case people with disability.^13^ While both roles were present in this project, peer researcher reflections are described separately.

In this paper, I reflect on the process of becoming an embedded researcher in a disability support service provider, and the challenges of this undertaking for both research and practice. Through reflecting on the ER literature and my lived experience, the reader is invited to consider the identified commonalities and the implications for future ER in a disability context. Embedded researchers for this paper are defined as people who work inside a host organization while maintaining affiliation with a university.^12^ Their purpose is implementing collaborative jointly owned research and fostering a mutually beneficial relationship.^1^

To situate my personal context, I have a professional background as a senior physiotherapist with experience using telehealth in hospitals, and undertook a role as an embedded PhD candidate within a disability support service provider organization focused on telepractice service co‐design. Telepractice is the delivery of predominantly clinical services such as allied health and nursing to people via telecommunications.^14^ In this project, it refers to real‐time video call therapy sessions between providers, people with disability, and their families. The project aimed to assess current telepractice service delivery and co‐design an improvement and implementation proposal in collaboration with people with disability and clinical staff over a three‐year period. Co‐design was the predominant method used to conduct the research, with sharing of power and decision‐making with the co‐designers (people with disability and staff) a priority throughout the implementation. The project included only one embedded researcher role which was funded by an Australian Government doctoral scholarship and a part‐time peer researcher (person with disability) role funded by the disability support service provider. Further information on the co‐design and outputs is published in Benz et al.^15^

The methods used in this reflection included a review of embedded researcher literature utilizing both database searches and reference list reviews of relevant articles to form a literature base to analyze in combination with personal reflection data. The personal reflection data collected during lived experience included research journal entries and other records of lived experience (photographs, audio recordings, drawings) maintained throughout the period in the role of embedded researcher. A reflexive thematic analysis of the personal reflection data and published literature was completed, and five themes formulated from the analytic process.^16^ The five themes discussed below include [1] a knowledge bridge, [2] considerations of positionality, [3] ethical complexity, [4] anticipating change, and [5] existing in the in‐between together.

## 2 A KNOWLEDGE BRIDGE

Reflecting upon the beginning of my journey as a PhD candidate embedded researcher, my mindset was that of being the link from research to the disability organization who had initiated the collaborative partnership. I was to funnel information toward the organization in return for willing research participants. However, I came to appreciate that the knowledge transfer was a process, and as Taylor et al.^2^ referred to ERs as “spanners” which spanned the gap, I came to reflect on embodying a kind of bridge. This change of mindset was marked by pivotal learning instances such as receiving guidance on the language of disability, such as the tension between the use of *identity first* (disabled person) versus *person first* language (person with disability) and the importance of being guided by each person's preference. My understanding of the importance of language was built through the privilege of learning from those who shared their experiences with me. I endeavored to share these lessons with the research members of the project team and acknowledged the importance of language in all academic publications. Lloyd^3^ reflected on valuing similar access to the language and world of child protection.

Co‐location was essential to what Cheetham et al.^1^ referred to as “organisational adhocracy” which involves creating incremental change when the opportunity presents. With the realms of academic literature largely inaccessible to most disability staff and consumers, I found essential groundwork to improve the understanding of the ER role and research more broadly often occurred in relaxed informal settings where open minds and curiosity had space to exist.

## 3 CONSIDERATIONS OF POSITIONALITY

Multiple ER reflections discuss the importance of considering positionality in their interactions.^2^, ^3^, ^4^, ^17^ The essence of ER is to be neither an insider nor an outsider researcher,^4^ thus my aim was to build trusting relationships with clinicians and people with disability, while using the insights they imparted in a sensitive and ethical way. In the context of a co‐design^18^ project, it was essential to maintain realistic contextual expectations of what the host organization could achieve to the co‐designers, while not over‐disclosing privileged knowledge of financial pressures and other constraints.

Another aspect of positionality discussed in the ER literature related to assuming the role of the “critical friend”^1^, ^17^, ^19^ or “critical niece.”^3^ The role of the critical friend provides an outsider perspective which assists in questioning and reflecting from an alternate viewpoint, and is posited to be a respected influence.^17^ Whereas the critical niece/nephew role is viewed as junior or new to an area in an effort to be nonthreatening and put staff at ease through an assumption of ignorance.^3^ In my experience, the tactical self‐depreciation of the critical niece role through an honest ignorance of disability practices needed to be balanced with the assertive influence of the critical friend. This was challenging and required significant emotional labor and maturity.

## 4 ETHICAL COMPLEXITY

The building of relationships, trust, and social capital which have repeatedly been described as foundational to successful ER,^2^, ^3^, ^4^ impart a level of responsibility and ethical consideration. The lack of clarity with regard to periods of embedded research to prepare for ethics approval^4^ and nondisclosure and information‐sharing agreements^3^ are relatively obvious areas for further progress in ER practice which at present remain without clear frameworks. While important, as reflected by Rowley^4^ in their work with vulnerable families, ethical considerations of ER are often far more complex and nuanced than current ethical approval processes can provide guidance with. When gathering contextually meaningful evidence,^6^ there is the potential to develop feelings of responsibility and conflicted loyalty particularly in contexts with marginalized populations.^4^, ^6^

Differing from traditional ethnographic research methods where ethical approval is sought to immerse in a context to collect data,^3^ this project involved participating in the context to focus on co‐design. Ethical approval did not explicitly include provisions for data gathered in the in‐between, before and after structured research sessions. Thus, posing the question as to how to manage these learnings. In these instances I predominantly relied on personal judgment regarding the intent by the person to consent for research use. This became particularly complex when interacting with people with disability, compounded by very limited available literature reflecting on direct contact with vulnerable populations such as Rowley.^4^ With the benefit of hindsight, I would proactively gain informed consent for data collected anecdotally in the in‐between, with an explicit description in participant information and consent forms. However, this is reliant on a person's awareness and proactive inclusion, therefore, I would also suggest there is a need for development of ethical approval processes which are designed to cater for participatory research formats such as co‐design and embedded research, which are increasing in frequency of use.

## 5 ANTICIPATING CHANGE

Building the foundations of trust and relationships to facilitate the bridging of knowledge involves certain conditions and preparations.^1^, ^3^, ^12^ The ER role is often novel, and certainly in my case required considerable procedural flexibility and negotiation of organizational processes. I additionally benefited from an organizational counterpart^2^ to vouch for my presence. This counterpart was able to open doors to meetings, introduce me to notable staff, advocated for my needs, and welcomed me to the organization. Contrastingly Duggan^17^ described feeling a nuisance, which I surmise would have been my experience without this crucial advocate.

In the context of a research tenure or PhD candidature as was undertaken by myself, Duggan,^17^ and Rowley,^4^ projects commonly takes 4+ years, which is an extended period time, thus increasing the risk of significant organizational changes. When social capital is your bartering tool,^3^ and staff or consumer buy‐in is your goal, frequent and dynamic staffing changes are extremely impactful and not uncommon.^2^, ^17^ In my case, of the 17 managerial roles in the department I was assigned to, there were 19 staffing changes across a three‐year tenure and only two unchanged positions. Additionally, the original primary supervisor who formed the partnership moved to a new tenure and was no longer able to directly support the industry and university partnership. This caused significant difficulty for relationship building and required extensive emotional labor to continually commit to rebuilding ER networks to progress the research.

## 6 EXISTING IN THE IN‐BETWEEN TOGETHER

When a high probability of change exists at the host organization,^17^ and your extended absence from academic circles impacts collegial connections, there is a truth that the in‐between can be a lonely place. Lloyd^3^ described feeling like she was living in her lanyard, always answering to multiple organizational requirements. Whereas, to continue the bridge metaphor I introduced earlier, I felt like I was neither here nor there, often with a vague feeling of not really fitting in anywhere. Much like a bridge I could not truly be on one side or the other and in my hardest times, feeling like no one would notice I was gone unless they had needed me.

Embedded researcher roles appear to be increasing in frequency, with 104 ERs responding to a survey by Mickan and Coates^20^ in Australia in 2019, and with this, an opportunity exists for greater support networks to be put in place to build networks of peer support. Taylor et al.^2^ demonstrated significant advantages in creating a community of practice for seven ERs involved in a climate science initiative in Southern African countries. Loneliness was commonly justified throughout my ER experience due to the isolating nature of a PhD tenure, however, I would have jumped at the opportunity for an ER community network, to reflect on challenging situations and brainstorm strategies. Evidence from the literature suggested that commonalities exist in embedded research across career stage (masters to post‐doctoral)^2^ and field of study,^1^, ^2^, ^3^, ^4^, ^6^, ^17^ therefore a community network could take the form of a university wide forum, or a network which is geographically located, that is, Australasian or European embedded research networks. On a smaller scale, creating more than one embedded role within a research project or academic department with planned opportunities to collaborate, reflect, and debrief.

I would also posit the need for greater awareness and learning opportunities for those in supervisory roles, to better understand the intricacies of being embedded.^12^ Although my supervisory team were greatly experienced, a more substantial appreciation of the unique characteristics of ER would have benefited all.

## 7 CONCLUSION

In conclusion, while the lived experience of ER in a disability host organization was invaluable in facilitating a successful co‐produced research project, significant avenues for improvement exist in terms of ethical frameworks, methodological guidance, and communities of support. Greater awareness is required that while it is a privilege to be invited to experience both a host organization and a university, even though you may experience both worlds, you won't ever belong to either. This position is unique to the ER role and while these are the reflections of a single ER in a disability organization, this and other reflections were well supported by ER literature across a wide range of sectors. Experiences of embedded research appear to transcend the discipline in which the research is being embedded, therefore university‐supported community of practice for all embedded postdoctoral, doctoral, or higher degree researchers would be beneficial.

## FUNDING INFORMATION

The article forms part of a PhD project funded through an Australian Government Research Training Programme (RTP) scholarship.

## CONFLICT OF INTEREST STATEMENT

The author declares that they have no competing interests.
